# *Brittle Culm 15* mutation alters carbohydrate composition, degradation and methanogenesis of rice straw during *in vitro* ruminal fermentation

**DOI:** 10.3389/fpls.2022.975456

**Published:** 2022-08-05

**Authors:** Siyu Yi, Xiumin Zhang, Jianjun Zhang, Zhiyuan Ma, Rong Wang, Duanqin Wu, Zhongshan Wei, Zhiliang Tan, Baocai Zhang, Min Wang

**Affiliations:** ^1^CAS Key Laboratory of Agro-Ecological Processes in Subtropical Region, National Engineering Laboratory for Pollution Control and Waste Utilization in Livestock and Poultry Production, Institute of Subtropical Agriculture, The Chinese Academy of Sciences, Changsha, Hunan, China; ^2^College of Animal Science and Technology, Guangxi University, Nanning, Guangxi, China; ^3^Institute of Bast Fiber Crops and Center of Southern Economic Crops, Chinese Academy of Agricultural Sciences, Changsha, China; ^4^Institute of Hunan Animal and Veterinary Science, Changsha, Hunan, China; ^5^State Key Laboratory of Plant Genomics, Institute of Genetics and Developmental Biology, The Innovative Academy of Seed Design, Chinese Academy of Sciences, Beijing, China

**Keywords:** rice straw, *Brittle Culm*, methane, hydrogen, rumen fermentation

## Abstract

*Brittle Culm 15* (*BC15*) gene encodes a membrane-associated chitinase-like protein that participates in cellulose synthesis, and *BC15* gene mutation affects cell wall composition in plant, such as cellulose or hemicellulose. The present study was designed to investigate the changes of carbohydrates composition in *bc15* mutant straw, and the resulting consequence on rumen fermentation, methanogenesis, and microbial populations (qPCR) during *in vitro* ruminal fermentation process. Two substrates, *bc15* mutant and wild-type (WT) rice straws, were selected for *in vitro* rumen batch culture. The first experiment was designed to investigate the kinetics of total gas and CH_4_ production through 48-h *in vitro* ruminal fermentation, while the second experiment selected incubation time of 12 and 48 h to represent the early and late stage of *in vitro* ruminal incubation, respectively, and then investigated changes in biodegradation, fermentation end products, and selected representative microbial populations. The *bc15* mutant straw had lower contents of cellulose, neutral detergent fiber (NDF) and acid detergent fiber (ADF), and higher contents of water-soluble carbohydrates, neutral detergent solubles (NDS) and monosaccharides. The *bc15* mutant straw exhibited a distinct kinetics of 48-h total gas and CH_4_ production with faster increases in early incubation when compared with WT straw. The *bc15* mutant straw had higher DM degradation, NDF degradation and total volatile fatty acid concentration at 12 h of incubation, and lower NDF degradation and CH_4_ production at 48 h of incubation, together with lower acetate to propionate ratio and ADF degradation and higher butyrate molar percentage and NDS degradation at both incubation times. Furthermore, the *bc15* mutant straw resulted in greater 16S gene copies of *F. succinogenes*, with lower 18S gene copies of fungi at both incubation times. These results indicated that the *BC15* gene mutation decreased fibrosis of cell wall of rice straw, enhanced degradation at the early stage of rumen fermentation, and shifts fermentation pattern from acetate to propionate and butyrate production, leading to the decreased volume and fractional rate of CH_4_ production. However, *BC15* gene mutation may enhance hardenability of cell wall structure of rice straw, which is more resistant for microbial colonization with decreased fiber degradation. Thus, this study modified rice straw by manipulating a cell wall biosynthesis gene and provides a potential strategy to alter degradation and CH_4_ production during *in vitro* ruminal fermentation process.

## Introduction

Rice straw is one of the major cropping by-products and can be used as a roughage source for ruminant animals (Zhang et al., 2020). However, rice straw cannot satisfy the maintenance needs of ruminants because of its high fiber content, low protein, and low energy level (Xu et al., 2018; Alemu et al., 2020). Thus, the resource of rice straw has not been effectively utilized although its yield is most abundant, and the import of alfalfa hay and other high-quality forages are increasing annually in China (Xu et al., 2018). Therefore, it has been a hot issue in the animal husbandry to find ways to reduce the fiber content of rice straw and increase its crude protein (CP) and non-fiber carbohydrate levels. In recent years, changing the nutritional components of rice straw has been widely investigated through physical, chemical, and microbial treatments (Zhang et al., 2018, 2020; Oskoueian et al., 2021). However, these technologies are usually limited in terms of cost or environment risk.

*Brittle Culm* (*BC*) genes are involved in cell wall formation of rice plants, and mutation of *BC* genes can modulate integrity of the cell wall (Li et al., 2019), which is accompanied by the changes of cell wall composition, such as cellulose or hemicellulose. The *Brittle Culm 15* (*BC15*) gene encodes a membrane-associated chitinase-like protein that participates in the synthesis of cellulose (Wu et al., 2012). The *BC15* gene mutation causes a reduction in cellulose content and mechanical strength and increases hemicellulose content without changed in plant growth (Wu et al., 2012). It seems that mutation of *BC15* gene can remodel cell wall through disturbing cellulose synthesis in rice straw, leading to change in cell wall structure and reduction in cellulose level. Such changes in cell wall composition of *bc15* mutant may alter nutritional composition of rice straw in ruminants, which has not been investigated before.

Carbohydrates are degraded to produce volatile fatty acids (VFA) in rumen microbial ecosystem for the host animals, accompanied with hydrogen (H_2_) production (Wang et al., 2016a). Ruminal H_2_ is mainly used by methanogens to produce methane (CH_4_; Janssen, 2010), which represents a loss of dietary energy and contributes to global anthropogenic greenhouse gas emissions (Wang et al., 2021a, 2022). Compared with forage fiber, non-fiber carbohydrates generally exhibit greater rate of fermentation and cause a shift in the VFA fermentation pattern from acetate to propionate or butyrate production, leading to a decrease in efficiency of H_2_ and CH_4_ production (Ma et al., 2015; Zhang et al., 2020). It seems that mutation of *BC15* gene can alter carbohydrate compositions of rice straw, which may lead to varied rumen fermentation and methanogenesis.

We hypothesized that *BC15* gene mutation possesses increased content of the soluble carbohydrates compared with the wild-type (WT) straw, which would alter rumen fermentation, methanogenesis, and microbial populations during ruminal fermentation process. To test this hypothesis, the *bc15* mutant and WT rice straws were employed for *in vitro* ruminal batch culture. We then firstly measured the kinetics of total gas and CH_4_ production through 48-h *in vitro* ruminal fermentation. Incubation time of 12 and 48 h were then selected to represent the early and late stage of *in vitro* ruminal incubation, respectively, and the second experiment was designed to investigate changes in biodegradation, fermentation end products, and selected representative microbial populations.

## Materials and methods

The experiment was approved (No. ISA W202101) by the Animal Care Committee, Institute of Subtropical Agriculture, the Chinese Academy of Sciences, Changsha, China.

### Substrates of *bc15* mutant and wild-type rice straw

The *bc15* mutant rice was isolated from tissue-cultured plants generated from the callus of the rice japonica cultivar Zhonghua 8 as described by Wu et al. (2012). These two genotypes of rice used in this study were grown in the experimental fields in Lingshui (Hainan Province, China) during the natural growing seasons. The wild-type (Zhonghua 8) and *bc15* mutant rice straw were harvested from mature plants after removing the grains under the same conditions and growing period.

The rice straws (not crushed) were dried in the sun under natural conditions. Then, approximately 100-g dried rice straws were crushed with a multi-functional pulverizer and screened through a 1-mm stainless steel flour sieve to ensure that the samples are homogeneous and have similar particle size, then the samples packed in hermetical plastic bags for chemical composition analysis, cell wall composition analysis, and *in vitro* ruminal fermentation. Beyond that, another 100-g dried rice straws from similar part of the stem in each rice straw were selected to a length of 2 cm, and then employed to investigate the changes in fiber structure after ruminal microbial degradation by using scanning electron microscopy.

### *In vitro* ruminal batch incubation and sampling

This study contained two *in vitro* experiments. The first experiment was designed to investigate the kinetics of total gas and methane (CH_4_) production after 48-h *in vitro* ruminal batch incubation. The second experiment was to compare the degradation and fermentation profiles of two straws at the early (12 h) and late (48 h) stages of *in vitro* ruminal incubation. Each experiment was conducted by a completely randomized block design, which included 3 runs with each treatment containing two fermentation bottles (replicates), and each run was incubated with mixed rumen fluid from 2 of 3 donor goats on different days. For the second experiment, each treatment had six fermentation bottles with two bottles for volatile fatty acids (VFA) and microbial samples, two bottles for scanning electron microscopy samples, and the other two bottles for substrates degradation measurement.

The *in vitro* ruminal batch culture was performed according to Wang et al. (2016b). Approximately 1.20 g of substrates were weighed into each of 135-ml serum bottles for the *in vitro* incubation. Rumen fluid was collected through permanent rumen cannula before morning feeding, which were fed by TMR diet containing the CP content of 127 g/kg of DM and the NDF content of 380 g/kg of DM. The rumen fluid was filtered through four layers of cheesecloth into a pre-warmed insulated bottle and taken to the laboratory. Then buffered rumen fluid was prepared by mixing 12-ml rumen fluid with 48-ml McDougall’s buffer (Cone and Becker, 2012), and then added into bottle under a stream of CO_2_ at 39.5°C. Bottles were immediately placed into an automatic incubation system (39.5°C, 55 r/min). Each bottle was connected to a pressure sensor, from which a signal operated a computer-controlled three-way solenoid valve. Venting pressure was set at 10 kPa, and vented gas was transferred to gas chromatographer (GC, Agilent 7890A; Agilent Inc., Palo Alto, CA, United States) to measure the CH_4_ concentration. Total gas production (GP) and CH_4_ production were calculated using the equations described by Wang et al. (2013).

Samples were collected at 12 and 48 h to represent the early and late stage of *in vitro* ruminal incubation, respectively. The 2 ml of liquid without visible particles were collected from two bottles and centrifuged at 12,000*g* for 10 min at 4°C. The supernatant (1.5 ml) was acidified using 0.15 ml of 25% (w/v) metaphosphoric acid, and stored at-20°C for analysis of VFA. The 1.5 ml of microbial samples were collected after intense shaking of the same two bottles to ensure that representative portions of liquid and particle fractions were included, and were immediately frozen in liquid N_2_ and stored at −80°C until DNA extraction. The pH was measured immediately with a portable pH meter (Starter 300; Ohaus Instruments Co. Ltd., Shanghai, China). Then, the 2-cm length of rice straw samples were taken from the other two fermentation bottles and dried in 65°C, then packed in hermetical plastic bags for scanning electron microscopy. Solid residues were filtered into pre-weighed Gooch filter crucibles from the other two bottles, dried at 105°C to determine degradation of incubated substrates.

### Sample analysis

The cell wall composition was determined according to the methods described by Huang et al. (2015). In brief, the feed samples were treated with 70% ethanol and a mixture of chloroform and methanol (1:1 v/v) twice to prepare alcohol insoluble residues (AIRs). After acid hydrolysis in 2 mol/l trifluoroacetic acid and derived into alcohol acetates, samples were analyzed by an Agilent 7,890 series GC equipped with a 5,975\u00B0C MS detector (Agilent, Palo Alto, California, United States). The cellulose content was quantified by anthrone assay using the remains after TFA treatment (Updegraff, 1969).

Contents of DM (method 945.15), OM (method 942.05) and CP (method 945.01, total *N* × 6.25) were analyzed according to methods of AOAC (2005). Neutral detergent fiber and ADF contents were determined with inclusion of a heat stable α-amylase and sodium sulfite, and were expressed as residual ash (Van Soest et al., 1991). Gross energy was determined by an isothermal automatic calorimeter (5E-AC8018; Changsha Kaiyuan Instruments Co, Changsha, China). The water-soluble carbohydrate (WSC) fraction was measured using the anthrone method (Yemm and Willis, 1954).

Alterations of fiber structure were obtained by field emission scanning electron microscopy (FESEM; model SU8010, Hitachi, Japan). Briefly, the similar part of the stem in each rice straw was selected and coated with gold before scanning (Zhang et al., 2018). The images representative of the average characteristics of each treatment group were screened with magnification of 1,000 or 5,000 times.

The VFA samples were recentrifuged at 15,000 g, and supernatants were collected to measuring the molar concentration of individual VFA by a GC (Agilent 7,890 A, Agilent Inc., Palo Alto, California, United States). Details of GC configurations were set according to the Wang et al. (2014). Molar percentage of individual VFA was then calculated based on their molar concentrations to better represent the fermentation pattern.

### DNA extraction and quantification of microbial groups

The microbial DNA was extracted by using a modified RBB + C methodology (Yu and Morrison, 2004) with sand beating according to Ma et al. (2020). The quality of the DNA extracts was assessed using agarose gel (0.8%) electrophoresis. Total DNA extracted was quantified with Nano Drop ND1000 (NanoDrop Technologies, Wilmington, DE, United States), and then stored at −80°C until further analyses.

Total DNA was then diluted to 10 ng/μl to quantify selected groups of microorganisms, including total bacteria, protozoa, fungi and methanogens, *Fibrobacter succinogenes, Ruminococcus albus, Ruminococcus flavefaciens, Selenomonas ruminantium, Prevotella ruminicola, Ruminobacter amylophilus, Butyrivibrio fibrisolvens, Methanobacteriales,* and *Methanobrevibacter* (Table 1). Quantitative PCR was performed according the procedures described by Jiao et al. (2014). Final absolute amounts of target groups or species were estimated by relating the C_T_ value to the standard curves and expressed as log_10_ copies/mL rumen contents.

**Table 1:** List of primers used for qPCR assay.

Microbial species	Primer sets (5′-3′)	Product size, bp	References
Protozoa	F: GCTTTCGWTGGTAGTGTATT;	223	Sylvester et al., 2004
	R: CTTGCCCTCYAATCGTWCT		
Fungi	F: GAGGAAGTAAAAGTCGTAACAAGGTTTC;	121	Denman and McSweeney, 2006
	R: CAAATTCACAAAGGGTAGGATGATT		
Bacteria	F: CGGCAACGAGCGCAACCC;	146	Denman and McSweeney, 2006
	R: CCATTGTAGCACGTGTGTAGCC		
Methanogens	F: GGATTAGATACCCSGGTAGT;	192	Hook et al., 2010
	R: GTTGARTCCAATTAAACCGCA		
**Selected groups of bacteria**			
*Fibrobacter succinogenes*	F: GTTCGGAATTACTGGGCGTAAA;	121	Denman and McSweeney, 2006
	R: CGCCTGCCCCTGAACTATC		
*Ruminococcus albus*	F: CCCTAAAAGCAGTCTTAGTTCG;	176	Koike and Kobayashiy, 2001
	R: CCTCCTTGCGGTTAGAACA		
*Ruminococcus flavefaciens*	F:GAACGGAGATAATTTGAGTTTACTTAGG;	132	Denman and McSweeney, 2006
	R:CGGTCTCTGTATGTTATGAGGTATTACC		
*Selenomonas ruminantium*	F: CAATAAGCATTCCGCCTGGG	138	Stevenson and Weimer, 2007
	R: TTCACTCAATGTCAAGCCCTGG		
*Prevotella ruminicola*	F:GAAAGTCGGATTAATGCTCTATGTTG	74	Stevenson and Weimer, 2007
	R: CATCCTATAGCGGTAAACCTTTGG		
*Ruminobacter amylophilus*	F: CTGGGGAGCTGCCTGAATG	102	Stevenson and Weimer, 2007
	R: GCATCTGAATGCGACTGGTTG		
**Selected groups of methanogens**			
*Methanobacteriales*	F:CGWAGGGAAGCTGTTAAGT;	343	Yu et al., 2005
	R:TACCGTCGTCCACTCCTT		
*Methanobrevibacter*	F: CCTCCGCAATGTGAGAAATCGC;	230	Huang et al., 2016
	R: TCWCCAGCAATTCCCACAGTT		

### Data analysis

The logistic-exponential model (Wang et al., 2011) was performed to analyze the kinetics of total gas production by using the Nonlinear Regression Analysis Program (NLREG, version 5.4; Sherrod, 1995), and expressed as follows:


$$GPt=VF\frac{1-\exp\left(-kt\right)}{1+\exp\left(b-kt\right)},$$

where *GP_t_* is the accumulated gas or CH_4_ production at time t (ml/g); *VF* is the final asymptotic gas or CH_4_ production (ml/g); *k* is the fractional rate of gas or CH_4_ production (/h); *b* is the shape parameter. Then, initial fractional rate of degradation at 0 h (FRD_0_, /h) was calculated using the following equation:

*FRD_0_* = *k* / [1 + exp (*b*)].

The net hydrogen production relative to the amount of total VFA produced (R_NH2_, mol/100 mol of VFA) was calculated using stoichiometric equations (Wang et al., 2014), and was expressed as follows:

R_NH2_ = [2(Ace + But + Isobut) − (Pro + Val + Isoval)] / total VFA.

where Ace, But, Pro, Val, Isobut, and Isoval indicate the concentrations (mmol/L) of acetate, propionate, valerate, isobutyrate, and isovalerate, respectively.

The neutral detergent solubles (NDS) content, hemicellulose content, and *in vitro* degradation was calculated according to the following equations (Zhang et al., 2020), and was expressed as follows:

NDS (g/kg of DM) = 1,000 − NDF (g/kg of DM).
hemicellulose (g/kg of DM) = NDF − ADF (g/kg of DM).
degradation (g/kg of DM) = [1 − (W_2_ × C_2_) / (W_1_ × C_1_)] × 1,000.

where C_1_ is NDS, NDF, or ADF content in the substrate before incubation; C_2_ is NDS, NDF, or ADF content in the residue after 12 or 48 h of incubation; W_1_ is DM weight of substrate before incubation; W_2_ is DM weight of residue after 12 or 48 h of incubation.

The values from replicated bottles for each run were averaged for statistical analysis. The data were then analyzed using a linear mixed model of SPSS 26.0 software (Chicago, IL, United States). The first analytic model included mutant (*n* = 2) as a fixed effect, and run (*n* = 3) as random effect. When sampling time was included, the second analytic model included mutant (*n* = 2) and interaction of mutant and sampling time as the fixed effect, sampling time (*n* = 2) as a repeated measurement, and run (*n* = 3) as the random effect. When significant interaction of mutant and sampling time was observed, statistical analysis was re-performed for each sampling time point by using the first model. Statistical significance value and a trend toward difference were set at levels of *p* ≤ 0.05 and 0.05 < *p* ≤ 0.10, respectively.

## Results

The *bc15* mutant straw had lower NDF (−7.33%) and ADF (−35.3%) contents, and higher CP (+97.4%) hemicellulose (+38.1%), WSC (+277%), and NDS (+20.5%) contents (Table 2). The *bc15* mutant straw had higher rhamnose (+17.2%, *p* < 0.001), fucose (+8.87%, *p* = 0.001), arabinose (+43.4%, *p* < 0.001), xylose (+24.8%, *p* < 0.001), mannose (+6.25%, *p* = 0.07) and galactose (+52.8%, *p* < 0.001) contents, and lower cellulose content (−37.3%, *p* < 0.001), although no difference was observed for glucose content between WT and *bc15* mutant straw (Table 3).

**Table 2:** Chemical composition of wild-type (WT) and *brittle culm 15* (*bc15*) mutant rice straws (expressed in g/kg of dry matter; *N* = 3).

Items	WT	*bc15*
OM	846	862
CP	23.0	45.4
NDF	737	683
ADF	456	295
Hemicellulose	281	388
WSC	12.2	46.0
NDS	263	317
Gross energy, MJ/kg DM	15.2	16.2

OM, Organic matter; CP, Crude protein; NDF, Neutral detergent fiber; ADF, Acid detergent fiber; WSC, Water-soluble carbohydrates; NDS, Neutral detergent solubles; GE, Gross energy.

**Table 3:** Cell wall composition of wild-type (WT) and *brittle culm 15* (*bc15*) mutant rice straws (expressed in g/kg of DM; *N* = 3).

Items	WT	*bc15*	SEM	Value of *p*
Rhamnose	2.15	2.52	0.033	<0.001
Fucose	1.24	1.35	0.014	0.001
Arabinose	24.9	35.7	0.59	<0.001
Xylose	145	181	2.1	<0.001
Mannose	2.40	2.55	0.049	0.07
Galactose	10.8	16.5	0.39	<0.001
Glucose	41.3	42.7	1.08	0.40
Cellulose	418	262	9.0	<0.001

SEM, Standard error of mean.

**Figure 1:**
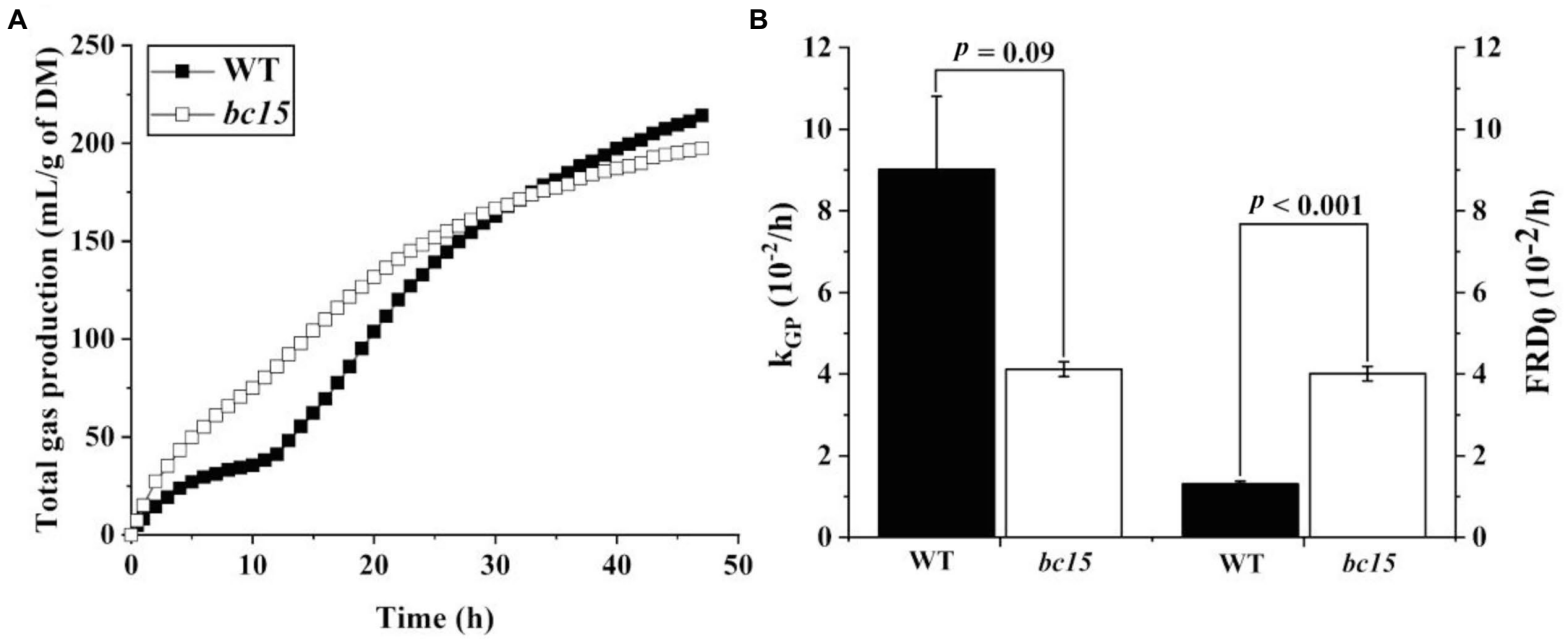
The kinetics of ruminal gas production **(A)** and its fractional rate of gas production (k_GP_, 10^−2^/h) and initial fractional rate of degradation at 0 h (**B**, FRD_0_, 10^−2^/h) of wild-type (WT) and *brittle culm 15* (*bc15*) mutant rice straws after 48-h *in vitro* ruminal incubation (*n* = 3). Data with error bars are expressed as mean ± standard error.

**Figure 2:**
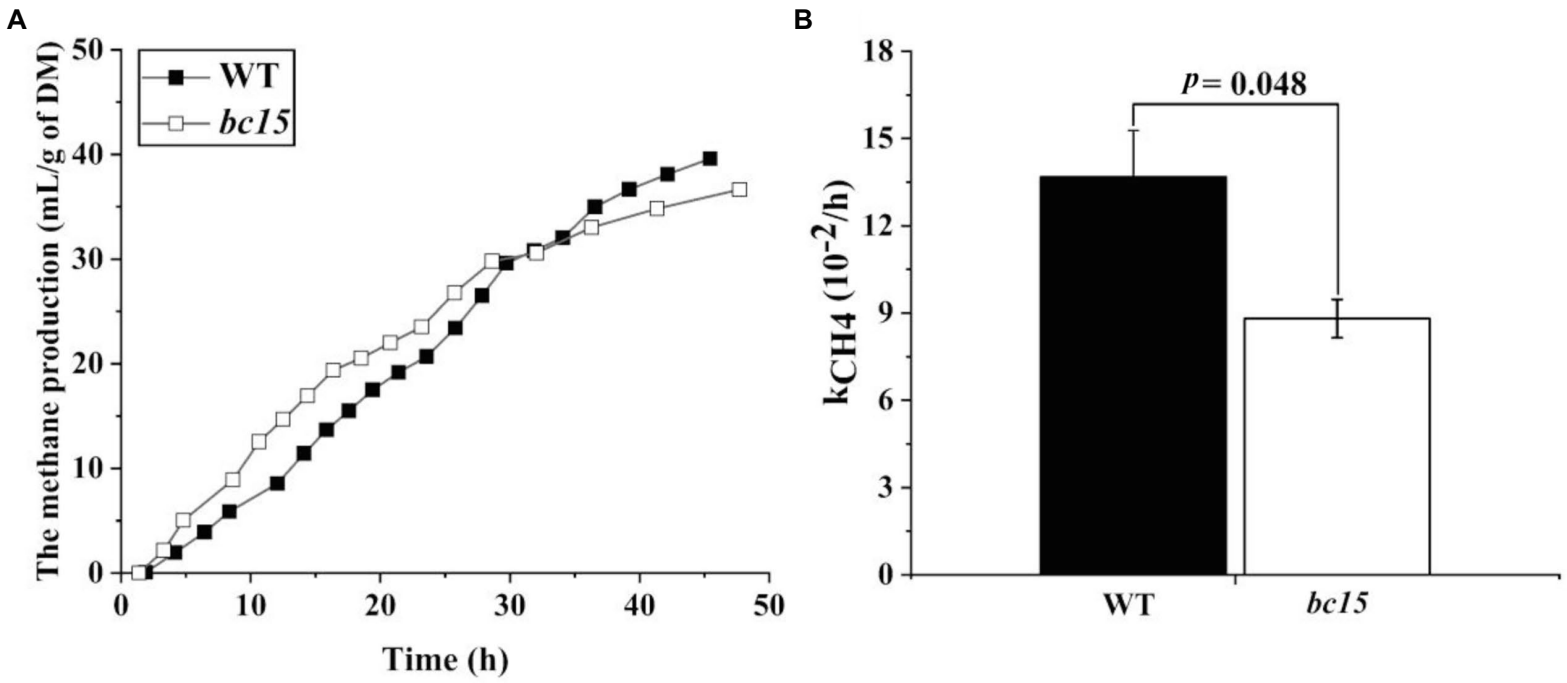
The kinetics of ruminal methane (CH_4_) production **(A)** and its fractional rate of CH_4_ production (k_CH4_, 10^−2^/h; **B**) of wild-type (WT) and *brittle culm 15* (*bc15*) mutant rice straws after 48-h *in vitro* ruminal incubation (*n* = 3). Data with error bars are expressed as mean ± standard error.

The *bc15* mutant straw displayed an altered kinetics of *in vitro* gas and CH_4_ production, with a faster increase in gas and CH_4_ production at first 24 or 30 h of incubation, and a reduction in gas and CH_4_ production after 36 h of incubation (Figures 1A, 2A). Further analysis of kinetics indicated that *bc15* mutant straw had greater initial fractional rate of degradation (*p* < 0.001), but lower fractional rate of CH_4_ production (*p* = 0.048, Figure 2B), together with a tendency of lower fractional rate of gas production (*p* = 0.09, Figure 1B). Significant interactions (*p* < 0.001) between mutant and time were observed for GP and CH_4_ production. The *bc15* mutant straw resulted in greater total gas (*p* < 0.001) and CH_4_ (*p* = 0.01) productions at 12 h of incubation, and lower total gas (*p* = 0.06) and CH_4_ (*p* = 0.01) productions at 48 h of incubation (Table 4).

**Table 4:** Gases production and degradation of wild-type (WT) and *brittle culm 15* (*bc15*) mutant rice straws after 12- and 48-h *in vitro* ruminal incubation (*n* = 3).

Items	12 h		48 h		SEM	Value of *p*^1^		
	WT	*bc15*	WT	*bc15*		Mutant	Time	Mutant × Time
Gases production, ml/g of DM								
GP	48.4	88.3^*^	218	204	2.69	0.001	<0.001	<0.001
CH_4_	4.10	11.9^*^	40.8	34.7^*^	1.80	0.24	<0.001	<0.001
Degradation, g/kg of DM								
DM	145	201^*^	476	472	12.9	0.04	<0.001	0.03
NDS	299	392	554	636	15.3	<0.001	<0.001	0.74
NDF	62.2	85.5^*^	431	380^*^	5.13	0.03	<0.001	<0.001
ADF	107	75.7^*^	451	344^*^	12.6	0.001	<0.001	0.02

GP, Total gas production; DM, Dry matter; NDS, Neutral detergent solubles; NDF, Neutral detergent fiber; ADF, Acid detergent fiber; SEM, Standard error of mean.

1 Mutant, Effects of *bc15* mutant and WT rice straws; Time, Effects of different incubation time; Mutant × Time, Interaction between mutant and incubation time.

* Indicates significant difference (*p* < 0.05) between bc15 mutant and WT rice straws at 12 or 48 h of incubation, when there was significant interaction between mutant and time (*p* < 0.05).

**Figure 3:**
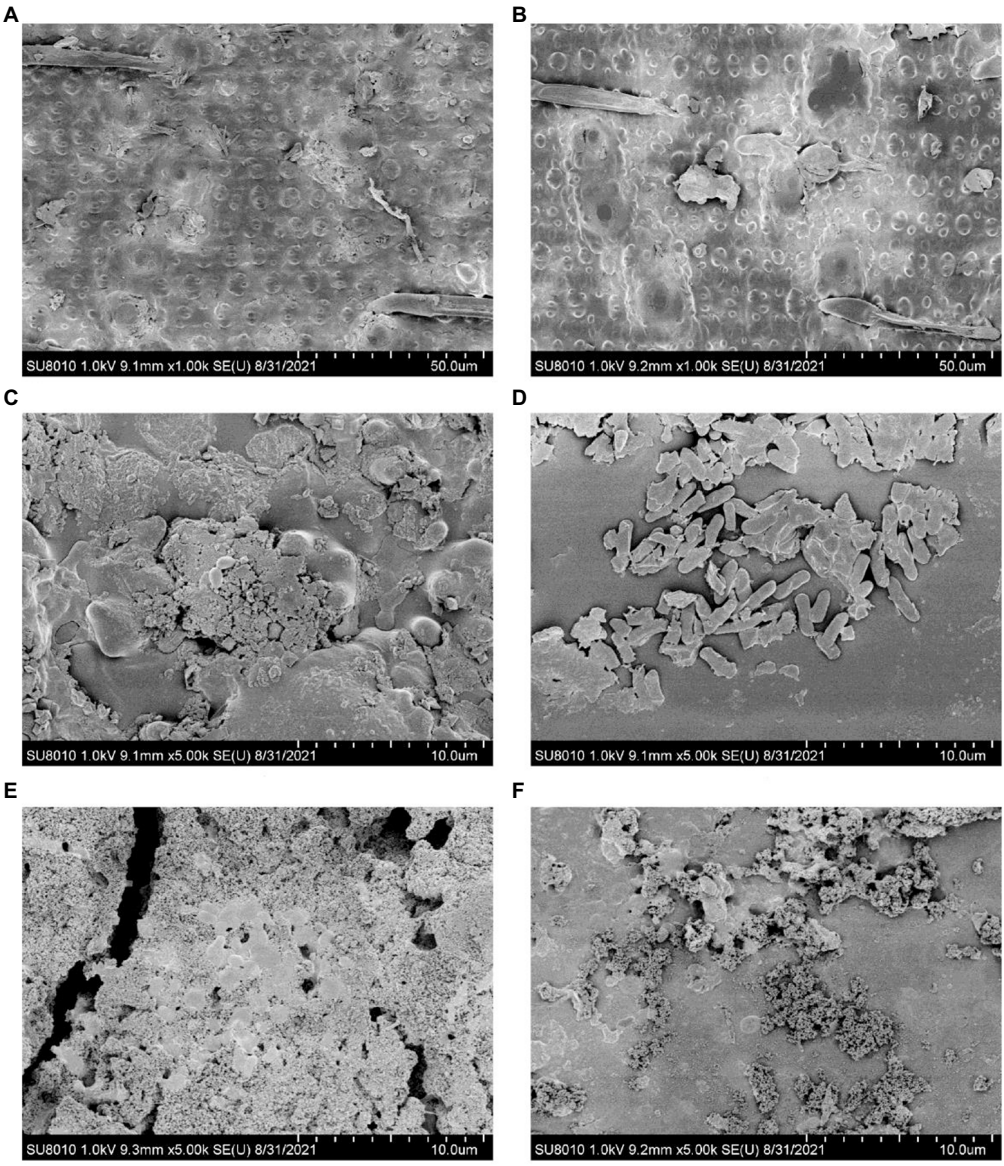
Scanning electron microscopy images of wild-type **(A,C,E)** and *brittle culm 15* **(B,D,F)** mutant rice straws before and after 12- and 48-h *in vitro* ruminal incubation.

The *bc15* mutant straw had greater NDS degradation (*p* < 0.001) and lower ADF degradation (*p* = 0.001) at both 12 and 48 h of incubation (Table 4). Interactions between mutant and time were observed for DM (*p* = 0.03) and NDF degradation (*p* < 0.001). The *bc15* mutant straw had higher (*p* < 0.05) DM and NDF degradation at 12 h of incubation, and lower NDF degradation (*p* = 0.01) at 48 h of incubation. The scanning electron microscopy analyses showed that a greater number of attached rumen microorganism were observed on *bc15* mutant straw at 12 h of incubation but with less small holes destroyed by rumen microorganism at 48 h of incubation when compared with WT straw (Figure 3).

Interactions (*p* < 0.05) between mutant and time were also observed for the changes of pH and VFA profile. The *bc15* mutant straw had lower pH (*p* < 0.001) and higher total VFA concentration (*p* = 0.03) at 12 h of incubation, although such differences were not observed (*p* > 0.1) at 48 h of incubation. The *bc15* mutant straw had lower (*p* = 0.002) acetate to propionate ratio at both 12 and 48 h of incubations, and higher (*p* = 0.008) propionate molar percentage at 12 h of incubation. The *bc15* mutant straw had lower (*p* = 0.004) acetate molar percentage and R_NH2_ (*p* = 0.03) and higher (*p* < 0.001) molar percentages of butyrate and valerate at both 12 and 48 h of incubations (Table 5).

**Table 5:** Fermentation characteristics of wild-type (WT) and *brittle culm 15* (*bc15*) mutant rice straws after 12- and 48-h *in vitro* ruminal incubation (*n* = 3).

Items	12 h		48 h		SEM	Value of *p*^1^		
	WT	*bc15*	WT	*bc15*		Mutant	Time	Mutant × Time
pH	6.76	6.62^*^	6.44	6.41	0.019	0.003	<0.001	0.02
Total VFA, mM	32.3	47.1^*^	82.4	79.1	4.51	0.15	<0.001	0.04
**Molar percentage of individual VFA, mol/100 mol**								
Acetate	67.6	64.8	67.7	64.3	0.78	0.004	0.78	0.63
Propionate	17.6	19.7^*^	23.8	23.6	0.26	0.007	<0.001	0.004
Butyrate	8.86	10.2	5.61	8.16	0.267	<0.001	<0.001	0.06
Isobutyrate	1.39	1.16^*^	0.80	1.05^*^	0.060	0.82	<0.001	0.004
Valerate	1.35	1.53	0.89	1.16	0.083	0.03	0.002	0.61
Isovalerate	3.17	2.54^*^	1.18	1.74^*^	0.249	0.88	0.001	0.03
Acetate to propionate ratio	3.84	3.29^*^	2.85	2.72	0.077	0.002	<0.001	0.02
R_NH2_ (mol/mol)	1.34	1.29	1.23	1.20	0.015	0.03	<0.001	0.29

R_NH2_, Estimated net hydrogen production relative to the amount of total VFA produced; SEM, Standard error of mean.

1 Mutant, Effects of *bc15* mutant and WT rice straws; Time, Effects of different incubation time; Mutant × Time, Interaction between mutant and incubation time.

* Indicates significant difference (*p* < 0.05) between bc15 mutant and WT rice straws at 12 or 48 h of incubation, when there was significant interaction between mutant and time (*p* < 0.05).

The further qPCR results showed that *bc15* mutant straw had greater (*p* = 0.002) 16S gene copy number of *F. succinogenes*, and lower 18S gene copy number of protozoa (*p* = 0.09) and fungi (*p* = 0.05) at both 12 h and 48 h of incubation. However, there was no difference (*p* > 0.1) in 16S gene copy number of bacteria, methanogens, *R. albus*, *R. flavefaciens*, *S. ruminantium*, *P. ruminicola*, *R. amylophilus*, *Methanobacteriales,* and *Methanobrevibacter* (Table 6).

**Table 6:** Select microbial groups (determined by RT-PCR) of wild-type (WT) and *brittle culm 15* (*bc15*) mutant rice straws after 12- and 48-h *in vitro* ruminal incubation, log_10_ (copies/ml; *n* = 3).

Items	12 h		48 h		SEM	Value of *p*^1^		
	WT	*bc15*	WT	*bc15*		Mutant	Time	Mutant × Time
Protozoa	10.1	9.90	8.37	8.13	0.118	0.09	<0.001	0.88
Fungi	7.86	7.68	8.62	8.11	0.150	0.05	0.004	0.30
Bacteria	11.7	11.7	11.6	11.6	0.07	0.74	0.21	0.76
Methanogens	10.1	10.1	9.97	9.91	0.163	0.85	0.32	0.87
**Selected bacteria**								
*Fibrobacter succinogenes*	9.01	9.63	9.06	9.27	0.092	0.002	0.14	0.06
*Ruminococcus albus*	8.52	8.60	7.85	7.69	0.197	0.73	<0.001	0.28
*Ruminococcus flavefaciens*	8.24	7.85	7.73	7.01	0.569	0.30	0.22	0.76
*Selenomonas ruminantium*	9.98	10.0	9.72	9.63	0.073	0.56	0.001	0.36
*Prevotella ruminicola*	10.0	10.1	10.1	10.0	0.15	0.99	0.92	0.69
*Ruminobacter amylophilus*	6.83	6.68	8.58	8.56	0.145	0.52	<0.001	0.67
**Selected methanogens**								
*Methanobacteriales*	9.34	9.26	9.16	9.12	0.195	0.68	0.30	0.89
*Methanobrevibacter*	9.22	9.34	9.20	9.08	0.145	0.99	0.34	0.43

SEM, Standard error of mean.

1 Mutant, Effects of *bc15* mutant and WT rice straws; Time, Effects of different incubation time; Mutant × Time, Interaction between mutant and incubation time.

## Discussion

Mutation of *BC15* gene has been reported to down-regulate cellulose biosynthesis in rice (Wu et al., 2012). The *bc15* mutant employed in our study exhibited higher CP, WSC, hemicellulose, and NDS contents, and lower NDF and ADF contents in rice straw. Furthermore, *bc15* mutant straw had lower cellulose content, and higher rhamnose, arabinose, xylose, mannose and galactose contents, in comparison with WT straw. Such changes in monosaccharides composition of cell wall are consistent with the study of Wu et al. (2012), which report that cellulose content is decreased through remodeling plant cell wall in *bc15* mutant rice straw. Furthermore, *bc15* mutant straw also exhibits higher CP content (45.4 vs. 23.0 g/kg of DM), which might be due to the activation of expression of specific genes in cellulose-deficient mutants (Raggi et al., 2015; Li et al., 2019), and such related mechanism needs further investigation.

The kinetics of 48-h total gas and CH_4_ production indicated a distinct ruminal fermentation process between *bc15* mutant and WT straws. The *bc15* mutant straw exhibited a higher gas and CH_4_ production than WT straw at early stage of incubation (<30 h). Such enhancement of fermentation at early stage of incubation was further supported by the greater FRD_0_, and was consistent with higher CP, WSC, NDS and monosaccharides content in *bc15* mutant straw. However, this was not the case for the late stage of incubation, as *bc15* mutant straw exhibited lower total gas and CH_4_ production than WT straw, which was consistent with lower NDF and ADF content in *bc15* mutant straw.

The *bc15* mutant straw consisted of changed carbohydrate compositions, and could alter substrates hydrolysis and degradation during *in vitro* ruminal fermentation, resulting in the significant interactions between mutant and time for substrates degradation. The *bc15* mutant straw had greater DM degradation at 12 h of incubation, but there was no difference in DM degradation at 48 h of incubation compared with WT straw. These results indicate that *BC15* gene mutation alters straw structure and/or composition, which enhances substrates degradation with increased initial fractional rate of degradation at early stage of incubation. Neutral detergent solubles, includes WSC, protein and ether extract, can be rapidly degraded by rumen microorganisms (Leiva et al., 2000). Previous study indicates elevated NDS content can promote degradation and gas production *in vitro* (Zhang et al., 2018, 2020). Forages with greater WSC content also have greater DM degradation rate and gas production *in vitro* (Amer et al., 2012). In our study, the elevated NDS and WSC content in *bc15* mutant straw contributed to the greater rate of degradation and gas production of rice straw at early stage of *in vitro* incubation, and enhanced NDS degradation at both incubation times.

Plant cell walls are mainly composed of cellulose, hemicellulose, lignin and pectin (Wang et al., 2016c), and its structure and composition may affect ruminal fiber digestibility (Zhang et al., 2020; Wang et al., 2021b). We observed significant interactions between mutant and time for NDF degradation with higher 12-h NDF degradation but lower 48-h NDF degradation and lower ADF degradation at both incubation times of the *bc15* mutant straw during *in vitro* incubation. Such enhancement of NDF degradation at early stage of incubation can be due to the greater monosaccharides (e.g., rhamnose, fucose, xylose, mannose, and galactose) content in *bc15* mutant straw, as monosaccharides in hemicellulose are usually more rapidly degraded in rumen than cellulose (Sun et al., 2007; Janssen, 2010). However, fermentation of *bc15* mutant straw exhibited the reduction in fiber degradation, which was supported by less small holes destroyed by rumen microorganism at 48 h of incubation. In cellulose-deficient mutants with defects in cell wall composition, plant cells often activate the expression of specific genes, such as peroxidase as a response to cell wall damage, which leads to the strengthening and hardening of cell walls and limits cell expansion (Raggi et al., 2015; Li et al., 2019). Such enhanced hardenability of cell wall structure in the *bc15* mutant straw likely causes greater difficulty to be degraded by rumen microorganisms compared with WT straw, leading to the reduction in fiber degradation.

Volatile fatty acid is mainly produced during ruminal carbohydrate fermentation, thus the VFA fermentation profile is greatly associated with carbohydrate composition. Cellulose is fermented to lesser propionate than acetate, whereas readily fermentable carbohydrates is fermented to less acetate but more butyrate and propionate (Janssen, 2010; Oba, 2011). The fermentation of *bc15* mutant straw resulted in greater butyrate molar percentage, and lower acetate molar percentage and acetate to propionate ratio at both 12 and 48 h of incubation times. The results are consistent with previous studies, which indicate that elevated WSC content in forages can increase butyrate or propionate molar percentage and decrease acetate to propionate ratio *in vitro* (Purcell et al., 2014; Rivero et al., 2020). It seems that increased content of soluble carbohydrates in the *bc15* mutant alters fermentation pathway of rice straw, leading to a less favorability of acetate production.

Hydrogen is produced during carbohydrate fermentation and is mainly utilized by methanogens to produce CH_4_ (Wang et al., 2016d; Martinez-Fernandez et al., 2017). The formation of acetate is associated with relative more H_2_ production, while the formation of propionate and butyrate are associated with net H_2_ consumption and relative less H_2_ production, respectively (Ungerfeld, 2020). Shifting fermentation pathway from acetate to propionate or butyrate production can be associated with a reduction in CH_4_ production (Wang et al., 2016a; Zhang et al., 2020). Significant interactions between mutant and time for CH_4_ production was observed. The *bc15* mutant straw had greater CH_4_ production at 12 h of incubation, which could be caused by greater extent of initial degradation. However, *bc15* mutant straw had lower CH_4_ production without any difference in DM degradation compared to WT straw at 48 h of incubation, indicating that the *BC15* gene mutation altered carbohydrate composition with less favorable methanogenesis, which was also reflected by the reduced R_NH2_ of *bc15* mutant straw. This result is consistent with previous studies, which found that fiber fermentation produces more CH_4_ than soluble carbohydrates (Pirondini et al., 2015; Bougouin et al., 2018; Zhang et al., 2020). Further studies are still needed to confirm the effect of *bc15* mutant straw on enteric CH_4_ emissions through feeding ruminant animals.

Efficient degradation of fibrous substrate can be achieved by a consortium of major cellulolytic groups. Fungi, protozoa, methanogens, and fibrolytic bacteria, such as *F. succinogenes*, *R. albus*, and *R. flavefaciens,* are the major cellulolytic microorganisms (Wang and McAllister, 2002; Koike and Kobayashi, 2009; Fuma et al., 2012). Fungi have been regarded as the primary colonizers and have the ability to destroy plant cell walls for subsequent attack by fibrolytic bacteria (Wang and McAllister, 2002; Zhang et al., 2007; Terry et al., 2019). In our study, the *bc15* mutant straw displayed lower 18S gene copy number of fungi, which is consistent to decreased ADF degradation and less fiber destruction observed during *in vitro* ruminal fermentation. Previous studies also indicate that increased fiber degradation can be closely associated with the increases in fungi population (Zhang et al., 2020; Wang et al., 2021b). However, the *bc15* mutant straw displayed greater 16S gene copy number of *F. succinogenes*. Although *F. succinogenes* is widely attributed to fiber degraders, it can also utilize different energy sources, such as structural and nonstructural carbohydrates (Ghali et al., 2017; Neumann et al., 2018). We propose that the fiber of *bc15* mutant straw would be more difficult for degradation with a reduction in population of major cellulolytic microorganisms.

## Conclusion

The *BC15* gene mutation altered carbohydrate compositions of rice straw with reduction in cellulose content and increase in hemicellulose, WSC, and NDS content. Such changes in carbohydrate composition altered the process of ruminal biodegradation, such as promoted degradation of rice straw at early stage (12 h) of *in vitro* rumen fermentation. However, cell wall of *bc15* mutant straw seems to be more resistant for microbial degradation, as demonstrated by lower fiber degradation and cellulolytic fungi population at 48 h of incubation. Furthermore, the *BC15* gene mutation also altered rumen fermentation of rice straw, which displayed shifted fermentation pattern from acetate production to propionate and butyrate production, leading to a reduction in efficiency of H_2_ production and 48-h CH_4_ production. Thus, the *BC15* gene mutation provides an excellent case for alter the nutritional components, degradation, and CH_4_ production of rice straw during *in vitro* ruminal fermentation process. Further researches are still needed to investigate the effects of mutant straws with more gene types on animal feeding.

## Data availability statement

The raw data supporting the conclusions of this article will be made available by the authors, without undue reservation.

## Ethics statement

The animal study was reviewed and approved by CAS Key Laboratory of Agro-Ecological Processes in Subtropical Region, National Engineering Laboratory for Pollution control and Waste Utilization in Livestock and Poultry Production, Institute of Subtropical Agriculture, The Chinese Academy of Sciences, Changsha, Hunan, 410125, China.

## Author contributions

SY conducted the experiments, analyzed the data, and wrote the initial manuscript. MW designed the research. MW and XZ performed funding acquisition. MW, XZ, and BZ reviewed the manuscript. BZ provided the experimental materials. JZ, ZM, and RW participated in experiment and sampling. DW and ZW performed formal analysis and investigation. ZT performed supervision and management. All authors contributed to the article and approved the submitted version.

## Funding

This work was supported by Strategic Priority Research Program of the Chinese Academy of Sciences (grant no. XDA26040203), National Natural Science Foundation of China (grant no. 31922080 and 32002204), Hunan Province Science and Technology Plan (2020NK2066 and 2022NK2021), the Science and Technology Innovation Program of Hunan Province (2021RC2102), China Agriculture Research System of MOF and MARA, Youth Innovation Promotion Association CAS (grant no. Y202078), and Open Fund of Key Laboratory of Agro-ecological Processes in Subtropical Region Chinese Academy of Sciences (grant no. ISA2021203).

## Conflict of interest

The authors declare that the research was conducted in the absence of any commercial or financial relationships that could be construed as a potential conflict of interest.

## Publisher’s note

All claims expressed in this article are solely those of the authors and do not necessarily represent those of their affiliated organizations, or those of the publisher, the editors and the reviewers. Any product that may be evaluated in this article, or claim that may be made by its manufacturer, is not guaranteed or endorsed by the publisher.
